# The Dipeptidyl Peptidase-4 Inhibitor Linagliptin Ameliorates High-fat Induced Cognitive Decline in Tauopathy Model Mice

**DOI:** 10.3390/ijms20102539

**Published:** 2019-05-23

**Authors:** Yuriko Nakaoku, Satoshi Saito, Yumi Yamamoto, Takakuni Maki, Ryosuke Takahashi, Masafumi Ihara

**Affiliations:** 1 Department of Neurology, Kyoto University Graduate School of Medicine, Kyoto 606-8507, Japan; yurikon@kuhp.kyoto-u.ac.jp (Y.N.); harutoma@kuhp.kyoto-u.ac.jp (T.M.); ryosuket@kuhp.kyoto-u.ac.jp (R.T.); 2 Department of Neurology, National Cerebral and Cardiovascular Center, Suita 565-8565, Japan; saitosa@ncvc.go.jp; 3 Research Fellow of Japan Society for the Promotion of Science, Tokyo 102-0083, Japan; 4 Department of Regenerative Medicine and Tissue Engineering, National Cerebral and Cardiovascular Center, Suita 565-8565, Japan; yumi.yamamoto@ncvc.go.jp

**Keywords:** dipeptidyl peptidase-4 inhibitors, high-fat diet, spatial reference memory, cerebral blood flow, tau

## Abstract

Vascular risk factors, such as type 2 diabetes mellitus (T2DM), are associated with the increased risk of Alzheimer’s disease. One of the common T2DM medications, dipeptidyl peptidase (DPP)-4 inhibitors, have a minimum risk for hypoglycemia and have recently been suggested to ameliorate β-amyloid pathology. However, conflicting results have been reported regarding the effects of DPP-4 inhibition on cognitive function and tau pathology. Thus, we investigated whether inhibiting DPP-4 affects tau pathology and cognition in a mouse model of tauopathy with hyperglycemia. Male mice overexpressing the P301S mutant human microtubule-associated protein tau gene (PS19) were fed either a low or high-fat diet. PS19 mice were then administered either linagliptin, a DPP-4 inhibitor, or vehicle, from 6 weeks to 8 months of age. Linagliptin-treated mice exhibited higher levels of glucagon-like peptide-1 and decreased fasting blood glucose, compared with the vehicle-treated mice at 8 months. Linagliptin treatment significantly restored spatial reference memory and increased cerebral blood flow without affecting phosphorylation levels of tau or endothelial nitric oxide synthase (eNOS) in the brain. Linagliptin may ameliorate HFD-induced cognitive worsening in tauopathy, at least partially, by increasing cerebral perfusion via the eNOS-independent pathway.

## 1. Introduction

Alzheimer’s disease (AD) is a common, progressive degenerative brain disease, leading to cognitive deficits, and accounts for 60–80% of global dementia cases [1]. The principle neuropathological hallmarks of AD are amyloid plaques and neurofibrillary tangles (NFTs), which are composed of aggregated β-amyloid (Aβ) peptide and hyperphosphorylated tau, respectively [2]. Many investigators have believed that Aβ is a critical factor for the initiation of the disease (as documented in the amyloid hypothesis) [3], and tau pathology is a secondary effect. Recent studies, however, suggest tau pathology plays a more important role than previously thought as it appears earlier in life, even before Aβ deposition in some individuals [4]. In frontotemporal dementia and parkinsonism linked to chromosome 17 (FTDP-17), mutations in microtubule-associated protein tau (MAPT) alone can cause neurodegenerative disease in the absence of Aβ [5]. Robertson et al. also demonstrated that reducing tau levels can prevent behavioral deficits in an AD mouse model without altering Aβ levels [6]. Indeed, another study found a strong correlation between the amount of neurofibrillary tangles in the neocortex and the degree of cognitive deficits in AD patients [7]. These data suggest tau, not amyloid, is the key player of neurodegeneration in AD. Nonetheless, the precise etiology of AD is still unclear and the relationship between Aβ and tau pathology needs to be determined. 

Type 2 diabetes mellitus (T2DM) is a major risk factor for neurocognitive disorders including AD [8]. In an aging society, an increasing number of people have both AD and T2DM. In the last few decades, the link between diabetes mellitus and AD has been recognized, sharing several common mechanisms, especially defective insulin signaling [9]. Insulin and its receptors are widely expressed in neurons and glial cells throughout the brain, suggesting insulin signaling may play a role in the control of cognition and neuronal function in brains. Several studies have reported significantly decreased neuronal insulin signaling in the cortex and hippocampus of AD cases [10]. Insulin resistance and deficiency also increase tau protein phosphorylation in both cultured neuronal cells [11] and in vivo models [12]. Indeed, a nutritional regimen based on a high-fat diet (HFD) has been shown to render the brain insulin-resistant [13] and exacerbate amyloidosis, tau phosphorylation and behavioral deficits in AD mouse models [14,15].

Some investigators refer to AD as type 3 diabetes or an insulin-resistant brain state [16,17]. Intracerebroventricular administration of the pro-diabetes drug streptozotocin in rats results in cognitive impairment with deficits in spatial learning and memory, brain insulin resistance and deficiency, and AD-type neurodegeneration [16]. Targeted exposure to a pro-diabetes drug can cause neurodegeneration that closely mimics AD pathology. It is therefore not surprising that T2DM treatment has been proposed as a potential therapy for AD [18]. Dipeptidyl peptidase-4 (DPP-4) is an enzyme that rapidly inactivates endogenous glucagon-like peptide-1 (GLP-1), an incretin hormone that induces insulin secretion [19]. Incretin-based therapies (GLP-1 analogs and DPP-4-inhibitors) are thus frequently used for the treatment of T2DM with a minimum risk for hypoglycemia [20]. GLP-1 receptors are abundantly expressed in the vascular endothelium and neurons in the central nervous system [21,22]. For this reason, GLP-1 has been suggested to have direct central action in the brain, as well as neurotrophic and neuroprotective effects, and thus, may have beneficial effects for the treatment of dementia [23].

Preclinical studies have pointed to incretin-based therapies as potential novel strategies in the treatment of AD [24]. In a transgenic mouse model of AD, peripherally-administered incretin reduced levels of important markers associated with AD neuropathology, including Aβ plaque load, microglial activation, and central insulin resistance [25,26]. Recently, Kosaraju et al. verified the neuroprotective activity of linagliptin in a 3xTg-AD mouse model (harboring triple PS1, APP and tau mutations) with concurrent β-amyloid and tau pathology [27]. The study demonstrated that linagliptin reduced both amyloid burden and tau phosphorylation. The mechanism by which phosphorylated tau was reduced may be an indirect effect of decreased Aβ. These preclinical studies have mainly focused on the therapeutic potential on amyloidosis-associated neurotoxicity; however, the effect on tau-associated pathology has not been fully elucidated. A tauopathy-specific model by Hansen et al. did demonstrate that a GLP-1 receptor agonist reduced neuronal phospho-tau load in hTauP301L mice but this study did not evaluate cognitive function [28]. In addition, a recent study presented a contradictory result, showing that sitagliptin aggravates tau phosphorylation in a rat model of T2DM and called for caution when administrating DPP-4 inhibitors to AD patients [29]. So far, no consensus has been made regarding the effects of DPP-4 inhibition on tauopathy-specific pathology and related cognitive dysfunction independent of amyloid pathology. 

In this study, we examined whether the DPP-4 inhibitor linagliptin affects tau pathology and cognition in a mouse model of tauopathy (PS19) challenged with HFD.

## 2. Results

### 2.1. Hyperglycemia by HFD was Ameliorated by Linagliptin Treatment

**Figure 1:**
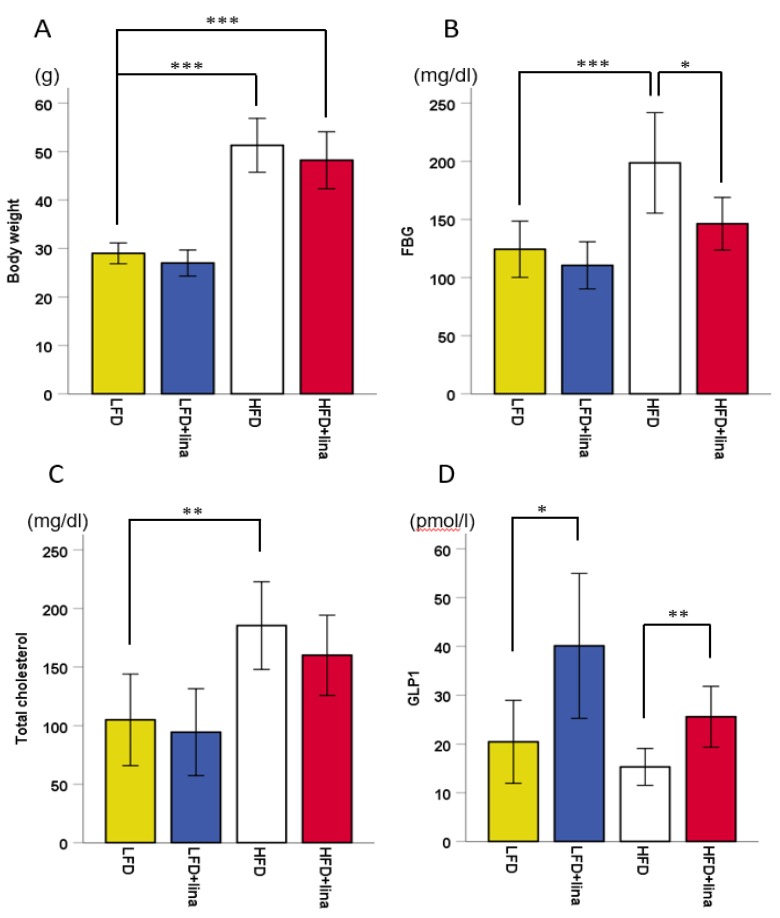
Effects of linagliptin (10 mg/kg BW/day) on (**A**) body weight, (**B**) fasting blood glucose (FBG), (**C**) total cholesterol, and (**D**) glucagon-like peptide-1 (GLP-1) in PS19 mice being fed a low-fat diet (LFD) or high-fat diet (HFD) for approximately 6 months. Mean ± SD. One-way ANOVA, followed by Bonferroni post hoc tests was used. Significant differences are indicated when * *p* < 0.05, ** *p* < 0.01, or *** *p* < 0.001.

After 5.5 months of HFD treatment, mice exhibited ~200% weight gain (*p* < 0.001, Figure 1A). Glucose and cholesterol levels were elevated in HFD-fed mice (*p* < 0.001 in Figure 1B and *p* < 0.01 in Figure 1C). Linagliptin-treated HFD-fed mice showed significantly increased blood GLP-1 levels compared to the vehicle-treated HFD-fed PS19 mice (Figure 1D), which may explain why fasting blood glucose levels in linagliptin-treated HFD-fed mice was significantly lower than vehicle-treated HFD-fed PS19 mice (*p* < 0.05, Figure 1B). 

### 2.2. Restoration of CBF in Linagliptin-Treated PS 19 Mice

**Figure 2:**
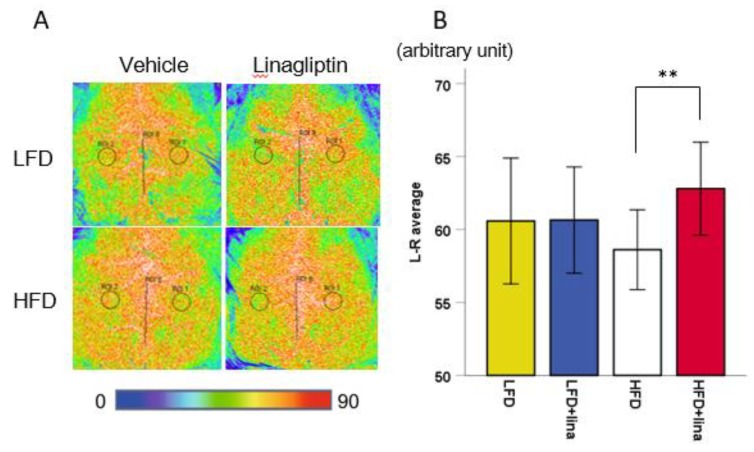
Effects of linagliptin (10 mg/kg BW/day) on cerebral blood flow (CBF). Linagliptin restored CBF reduction. (**A**) Representative images showing CBF measured by laser speckle flowmetry in vehicle-treated mice (left) and linagliptin-treated mice (right) at 7 months of age. (**B**) A histogram showing CBF. Error bars indicate ± SD, ** *p* < 0.01.

Impairment of cerebral circulation has a significant role in the onset and progression of cognitive dysfunction in AD patients and animal models [30]. To assess the vascular effect of linagliptin in PS 19 mice, CBF was measured at 7 months of age using laser speckle flowmetry. Linagliptin-treated HFD-fed mice exhibited significantly increased CBF compared with vehicle-treated HFD-fed PS19 mice (*p* < 0.01, Figure 2). 

### 2.3. Normalization of Spatial Reference Memory Impairment in HFD-fed PS19 Mice .

**Figure 3:**
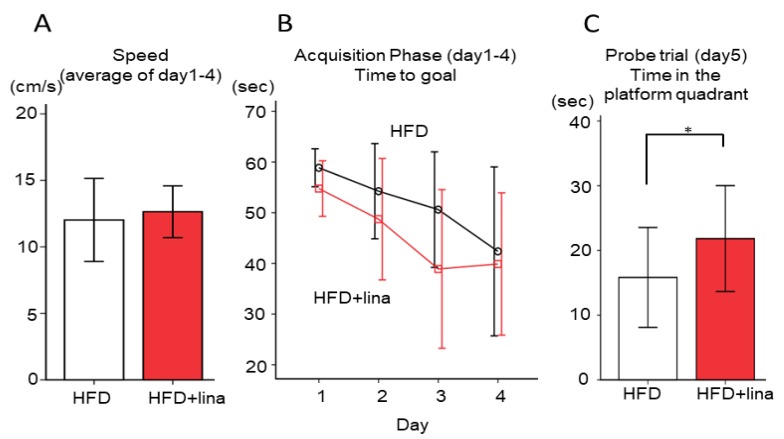
Effects of linagliptin on cognitive function as assessed by the Morris water maze test. (**A**) Swimming speed was unaffected, (**B**) while the time course of escape latency (time to goal) in the acquisition phase was reduced more in the linagliptin-treated group than in the vehicle-treated group. (**C**) The time spent in the target quadrant in the probe trial was also significantly higher in the linagliptin-treated group. Error bars indicate ± SD, * *p* < 0.05.

We evaluated whether linagliptin (10 mg/kg BW/day) treatment affected spatial learning and reference memory impairment by the Morris water maze test at 8 months of age. Linagliptin-treated HFD-fed PS19 mice demonstrated a gradual improvement in learning during acquisition trials (days 1–4) and exhibited significantly shorter escape latencies than vehicle-treated HFD-fed PS19 mice (*p* < 0.05, Figure 3B), while motor function was not affected (Figure 3A). During the probe trial on day 5, the time spent in the platform quadrant in the linagliptin-treated HFD-fed group was significantly greater than those in the vehicle-treated HFD-fed group (*p* < 0.05, Figure 3C). These results indicated that linagliptin restored spatial reference memory impairment in HFD-fed PS19 mice.

### 2.4. Immunohistochemistry

**Figure 4:**
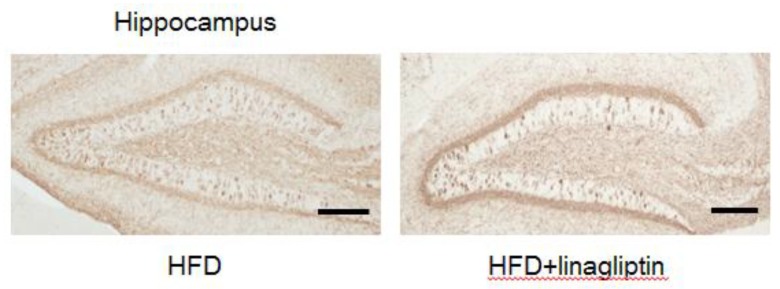
Immunohistochemical assessment of phosphorylated tau pathology in the hippocampus after the administration of linagliptin. Representative images of phosphorylated tau staining in vehicle-treated (left) and linagliptin-treated mice (right) at 8 months of age. Bar = 100 µm

To evaluate whether improvement in cognitive function resulted from decreased tau deposition by linagliptin, mouse brains were stained with anti-phospho-tau antibody (clone AT8). Figure 4 shows representative images of brain sections from vehicle and linagliptin-treated PS19 mice. Phosphorylated tau was not reduced in the hippocampus of linagliptin-treated PS19 mice.

### 2.5. Western Blotting

**Figure 5:**
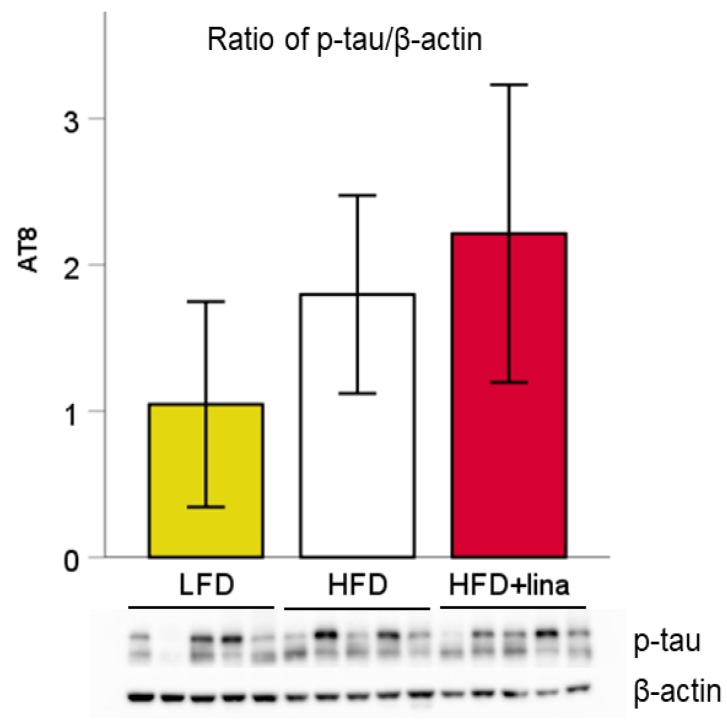
Effects of linagliptin (10 mg/kg BW/day) on tau phosphorylation in PS19 mice at 8 months of age. Representative Western blot for ratio of phosphorylated tau (p-tau)/β-actin. Values are presented as the mean ± SD (*n* = 5 for each group).

We also quantified the amount of phosphorylated tau in vehicle or linagliptin-treated mice by Western blotting. No significant change in phosphorylated tau was observed between linagliptin-treated and vehicle-treated HFD-fed PS19 mice (Figure 5). Although an inbred strain was used in this study, the amount of phosphorylated tau showed a large variation within the group, which may explain why no statistical difference was observed even between LFD- and HFD-fed mice. Linagliptin seems to have affected the cognitive function and CBF levels independently of the status of tau phosphorylation. 

**Figure 6:**
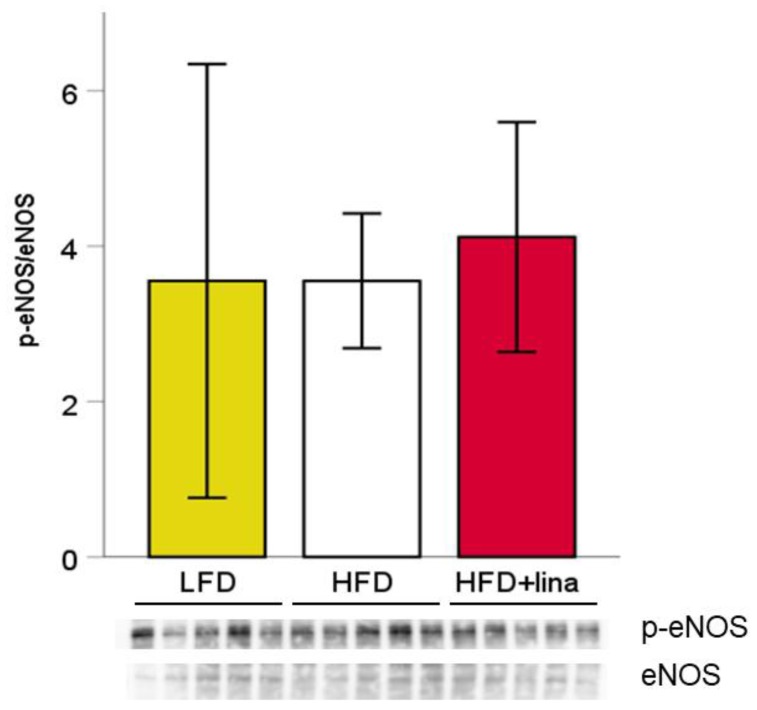
Effects of linagliptin (10 mg/kg BW/day) on tau phosphorylation in PS19 mice at 8 months of age. Linagliptin did not significantly affect the ratio of cerebral phosphorylated eNOS/eNOS. Values are presented as mean ± SD (*n* = 5 for each group).

To investigate whether linagliptin improved CBF via protection of vascular endothelial function, we quantified the amount of endothelial nitric oxide synthase (eNOS) and phosphorylated eNOS in vehicle or linagliptin-treated mice by Western blotting. However, no significant change in phosphorylated eNOS was observed between linagliptin-treated and vehicle-treated HFD-fed PS19 mice (Figure 6).

## 3. Discussion

The purpose of the present study was to evaluate whether linagliptin improves or aggravates cognitive impairment and tau pathology in a mouse model of tauopathy (PS19). PS19 mice used in this study express mono-transgenic P301S mutant human tau and develop filamentous tau lesions at 6 months of age, subsequently progressing to tau accumulation in association with neuronal loss by 9–12 months of age [31]. As this model does not express amyloid pathology, it is considered ideal for tauopathy-specific conditions. No studies have previously examined how DPP-4 inhibitors affect tau pathology and memory deficits in tauopathy models. In the present study, we showed that linagliptin reversed HFD-induced cognitive decline and increased CBF in PS19 mice. Unexpectedly, however, phosphorylation of tau was neither increased nor decreased, as reported in previous studies [27,29]. Such contradictory results may stem from differences in animal models, medication and experimental design. Kosaraju et al. demonstrated improved tau pathology in 3xTg-AD mice administered with linagliptin orally (5, 10, and 20 mg/kg) for 8 weeks starting from 9 months of age [27]. However, the study reported aggravation of tau pathology, used a 21 week-old T2DM model rat and administered sitagliptin orally (100 mg/kg/day) for 12 weeks [29]. Since NFT pathology spreads long before cognitive symptoms appear, the oral administration of linagliptin (10 mg/kg/day) in this study started from a much younger age (6 weeks) and for a longer period of time (6.5 months). The fact that our PS19 mice were under additional hyperglycemic stress to induce cognitive decline also complicated the direct comparison of our data with that of previous studies. Nevertheless, our report may provide important insights for the effectiveness and long-term safety of DPP-4 inhibitors in dementia due to tauopathy. 

Considering the lack of improvement in tauopathy by linagliptin, we concluded that the restored cognitive function was mainly attributed to increased CBF. Indeed, previous studies have reported that DPP-4 inhibitors improve vascular function via endothelium-dependent vasodilatory effects. Hasegawa et al. showed that linagliptin ameliorated cognitive impairment, which was associated with an increase in CBF, at least in part mediated by an increase in cerebral phospho-eNOS [30]. However, we did not detect any significant changes in phosphorylated eNOS between linagliptin- and vehicle-treated HFD-fed mice. Several recent studies have suggested that the inhibition of DPP4 exerts beneficial pleiotropic effects aside from their anti-hyperglycemic properties. Linagliptin has been shown to improve cerebrovascular dysfunction observed in diabetes through anti-inflammatory and vasodilatory effects in a glucose-independent manner [32]. Alternatively, linagliptin may restore cerebrovascular function via the regulation of hyper-reactivity to endothelin 1 and Toll-like receptors 2 expression [33]. Treatment with sitagliptin was reported to reverse memory impairment in HFD-fed mice through reduced oxidative stress and enhanced neurogenesis [34]. In our study, while treatment with linagliptin in PS19 mice fed with a high-fat diet was able to restore cerebral perfusion, there was no similar improvement in cerebral perfusion in PS19 mice fed with a standard diet.

Several limitations in this study should be addressed. Firstly, we conducted behavioral and biochemical analysis at 8 months only, while PS19 mice are reported to develop tau accumulations progressively by 9–12 months of age. Further studies are needed to elucidate whether the amount of phosphorylated tau or phosphorylated eNOS is increased in older age and if linagliptin is more effective at this time point. Secondly, we used a set dose of 10 mg/kg BW/day in this study. Considering that the previous report using 3xTg-AD mice demonstrated dose-dependent effects of linagliptin for cognitive function and tau phosphorylation [27], varying doses of linagliptin should be tested in future studies. Finally, we could not identify the exact mechanism of CBF increase by linagliptin. Further studies are warranted to elucidate the underlying mechanisms of the data presented here. 

## 4. Materials and Methods 

### 4.1. Animals

PS19 transgenic mice expressing human (1N4R) with P301S mutation driven by mouse prion protein promoter (Prnp) [31] were purchased from Jackson Laboratories. PS19 mice display age-related NFTs from 6 months of age, neuron degeneration by 8 months of age, and subsequently behavioral deficits. These mice were maintained on a C57BL/6J background. All animal protocols were approved by the Institutional Animal Care and Use Committee at the National Cerebral and Cardiovascular Center (Permit Number: 15045, 16043, 1 April 2015), and were performed in accordance with the Guidelines for Proper Conduct of Animal Experiments established by Science Council of Japan.

### 4.2. Study Design

**Figure 7:**
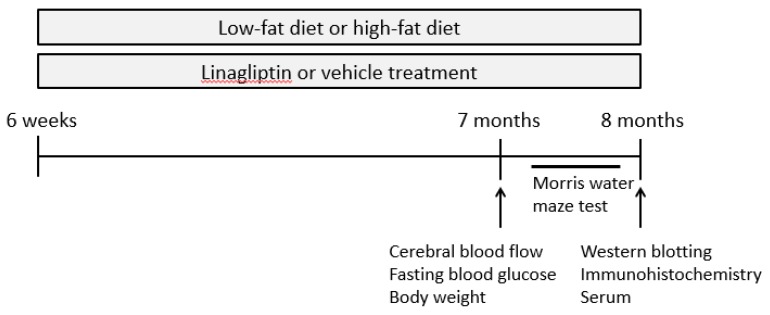
Scheme of experimental procedure.

Male heterozygous PS19 mice aged 6 weeks were randomly assigned to two groups and received either vehicle or linagliptin (100 mg/L, kindly provided by Boehringer Ingelheim) in their drinking water, at 10 mg/kg BW/day, which is frequently used and reported to be the most effective dose in a previous study [35]. Both groups were fed either with a low-fat diet (LFD; caloric composition, 10% fat, 70% carbohydrate, and 20% protein, Research Diet, Inc, New Brunswick, NJ, USA; vehicle, *n* = 15; linagliptin, *n* = 17) or high-fat diet (HFD; caloric composition, 60% fat, 20% carbohydrate, and 20% protein, Research Diet, Inc, New Brunswick, NJ, USA; vehicle, *n* = 15; linagliptin, *n* = 17) in order to induce hyperglycemia. At 7 months of age, evaluations of body weight, fasting serum glucose, and cerebral blood flow (CBF) were conducted. At 8 months of age, spatial reference memory was assessed by the Morris water maze test (MWM). After behavioral testing, mice were sacrificed under deep anesthesia to obtain a blood sample (Figure 7). PS19 mice were then perfused transcardially with 0.01 mol/L phosphate buttered saline (PBS). Brains (*n* = 10 per group) were immediately removed and divided along the sagittal line into two hemispheres for Western blotting and immunohistochemistry.

### 4.3. Blood Testing

Blood samples for blood glucose level were collected from the tail vein after 6-hour fasting. Blood glucose level was measured by the glucose dehydrogenase method using Glutest Neoα (Sanwa Kagaku Kenkyusho, Japan). For the measurement of GLP-1 and total cholesterol, serum was prepared from blood samples collected from the inferior vena cava during animal sacrificing. Active GLP-1 concentrations in plasma were measured by the Active form Assay kit-IBL (Immuno-Biological Laboratories, Gumma, Japan). Plasma total cholesterol level was measured by automated clinical chemistry analyzer Fuji dri-chem 7000 with Fuji dri-chem slide TCHO-PIII (Fujifilm, Tokyo, Japan). 

### 4.4. Measurement of Cerebral Blood Flow

Relative CBF of Tg-PS19 mice was measured by laser speckle flowmetry (Omegazone-2, Omegawave, Fuchu, Japan), as previously reported [36]. Anesthesia was induced with 2%, and maintained with 1.5% isoflurane. The scalp was removed by midline incision to expose the skull throughout CBF evaluation. CBF was measured in identically-sized, round regions of interest (1 mm in diameter), located bilaterally at 1 mm posterior and 2 mm lateral from the bregma, corresponding to regions around Heubner’s anastomoses. Average CBF values in the bilateral hemispheres were recorded. 

### 4.5. Behavioral Analysis: Morris Water Maze Test

After 6.5 months of linagliptin treatment, spatial reference learning and memory were evaluated by the Morris water maze test, consisting of a circular pool (diameter, 120 cm; depth, 40 cm) and a set of video analysis systems (EthoVision XT5; Noldus, Wageningen, Netherlands), as previously described [36]. During training, a platform (10 cm in diameter) was submerged 1 cm below the water surface in the center of one quadrant of the pool (target quadrant). During the first 4 days, all mice were given four trials per day with a 30-minute interval between attempts (acquisition phase). Mice were placed at the starting position (the quadrant adjacent to the target) and released into the water. For each trial, the mouse was given 60 seconds to reach a hidden platform. The latency to reach the platform (escape latency), swim distance, and mean swimming speed were recorded. During the probe trial on the fifth day, mice were allowed to swim without the platform for 60 seconds to search for it. The duration of swimming in each quadrant was recorded.

### 4.6. Western Blotting

The left hemisphere was cut coronally into 2-mm-thick slices (bregma +2 mm to +4 mm) and homogenized in RIPA buffer containing protease inhibitor cocktail and phosphatase inhibitor cocktail (all from Nacalai Tesque, Kyoto, Japan). Ten micrograms of total protein was resolved by 10% SDS-PAGE, transferred onto Immobilon-P PVDF membranes (Millipore), and analyzed by Western blotting. Primary antibodies used in this study included AT8 mouse monoclonal anti-human tau pSer202/Thr205 (Thermo Fisher Scientific, Rockford, IL, USA), rabbit polyclonal eNOS (Cell Signaling), rabbit monoclonal phospho-eNOS (Ser1177) (Cell Signaling), and rabbit polyclonal β-actin (Abcam). The densitometric value of each band was quantified using NIH-based Image-J software. 

### 4.7. Immunohistochemistry

Dissected right hemisphere samples (*n* = 10 per group) were post-fixed in 4% paraformaldehyde in 0.1M phosphate buffer overnight, embedded in paraffin, and sliced into 6-μm-thick sagittal sections starting from the position 1 mm lateral from the midline using a microtome (Leica, Nussloch, Germany). Sections were treated for heat-mediated antigen retrieval in 0.01M citrate buffer (pH 6.0) for 10 min. Endogenous peroxidase activity was inactivated by 3% hydrogen peroxide in methyl alcohol for 10 minutes. Immunostaining with mouse monoclonal anti-human tau pSer202/Thr205 antibody (Thermo Fisher Scientific) was performed with Vector M.O.M. Immunodetection Kit (Vector Laboratories). Visualization of bound peroxidase was achieved by incubation in TBS containing 0.05% diaminobenzidine (DAB) and 0.045% hydrogen peroxide. 

### 4.8. Statistical Analysis

All values are expressed as means ± SD unless stated otherwise in the figures. Individual comparisons were analyzed by a Student’s *t* test or ANOVA followed by post hoc Turkey tests. Differences with a probability value of *p* < 0.05 were considered statistically significant in all analyses. Statistical analysis was conducted using SPSS Statistics version 23.0 (IBM, Armonk, NY, USA).

## 5. Conclusions

Our investigation indicates that the administration of linagliptin improves HFD-induced cognitive dysfunction in PS19 mice, rather than aggravating tau pathology. The present work thus suggests DPP-4 inhibitors may be suitable in the management of T2DM patients at risk of cognitive impairment, as a result of its beneficial effects on cerebral perfusion and cognitive function.

## Abbreviations

T2DM	Type 2 diabetes mellitus
DPP-4	Dipeptidyl peptidase-4
HFD	High-fat diet
LFD	Low-fat diet
AD	Alzheimer’s disease
Aβ	β-amyloid
NFT	neurofibrillary tangle
GLP-1	glucagon-like peptide-1
FTDP-17	frontotemporal dementia and parkinsonism linked to chromosome 17
CBF	cerebral blood flow
MAPT	microtubule-associated protein tau
Prnp	prion protein promoter
PCR	polymerase chain reaction
MWM	Morris water maze test
NO	nitric oxide
EDHF	endothelium-derived hyperpolarizing factor
eNOS	endothelial nitric oxide synthase
PBS	phosphate buttered saline
RIPA	radio-immunoprecipitation assay
PVDF	polyvinylidene difluoride
DAB	diaminobenzidine
